# Oppression‐based stressors, posttraumatic stress symptoms, and self‐injurious thoughts and behaviors among a national sample of queer adolescents of color

**DOI:** 10.1002/jts.70069

**Published:** 2026-04-22

**Authors:** Tara R. Sullivan, Marianna Amato, Jeremy T. Goldbach, Ethan H. Mereish

**Affiliations:** ^1^ Lavender Lab, Department of Psychology University of Maryland College Park Maryland USA; ^2^ Department of Education Studies University of California San Diego, La Jolla California USA; ^3^ Brown School Washington University in St. Louis St. Louis Missouri USA

## Abstract

Oppression‐based stressors (OBS; e.g., heterosexism‐based stressors) are associated with a higher risk of trauma‐related symptoms and self‐injurious thoughts and behaviors (SITB) for queer (e.g., lesbian, gay, bisexual) adolescents. However, prior research has yet to examine posttraumatic stress symptoms (PTSS) and SITB in the context of the unique, intersectional OBS experienced by queer adolescents who are also Black, Indigenous, and/or people of color (BIPOC). This study examined the associations among intersectional OBS, PTSS, and SITB and tested identity outness as a potential moderator of these associations. A national sample of 1,009 queer BIPOC adolescents (*M*_age_ = 15.97 years, *SD* = 0.93; 62.0% cisgender girls, 50.1% plurisexual) was recruited online. After accounting for sociodemographic characteristics and overall perceived stress, OBS, β = .08, and identity outness, β = .08, were significantly associated with higher PTSS. OBS and identity outness were also associated with a higher likelihood of endorsing all three suicide outcomes (suicidal ideation, suicide planning, and suicide attempts), adjusted odds ratios (a*OR*s) = 1.35 and 1.55, respectively, as well as a higher likelihood of nonsuicidal self‐injury, a*OR*s = 1.22 and 1.21. Identity outness did not serve as a moderator in the associations among OBS, PTSS, and SITB. The findings suggest that intersectional OBS may serve as a risk factor for PTSS and severe levels of SITB among queer BIPOC adolescents. Further research is needed to identify the unique role of outness among queer BIPOC adolescents and additional factors that may protect against the negative mental health outcomes associated with OBS.

Queer (e.g., lesbian, gay, bisexual) adolescents have an increased risk of trauma exposure, posttraumatic stress symptoms (PTSS), and probable posttraumatic stress disorder (PTSD) as compared to heterosexual adolescents (Roberts et al., 2012). Queer adolescents also report elevated rates of self‐injurious thoughts and behaviors (SITB) and nonsuicidal self‐injury (NSSI) compared to their heterosexual peers, and traumatic stressors are a notable risk factor for NSSI and other SITB among queer adolescents (Ellis & Tate, 2022; Rogers & Taliaferro, 2020). Moreover, queer adolescents who are racially/ethnically minoritized (i.e., Black, Indigenous, and/or people of color [BIPOC]) are more likely to report SITB than White queer adolescents (Bostwick et al., 2014). Thus, an intersectional approach is imperative to understand the nuances of how PTSS and SITB manifest among queer BIPOC adolescents.

Intersectionality frameworks posit that one's social identities and experiences of oppression, power, and privilege are shaped by numerous interactions between systems of oppression (e.g., heterosexism, racism; Crenshaw, 1989). According to the intersectional oppression‐based stress framework, oppression‐based stressors (OBS) are perpetuated by intersecting oppressive systems (e.g., heterosexism, cissexism, racism) at multiple levels (Mereish, 2024). OBS serve as distressing experiences that inflict harm and shame, disrupt important developmental processes, and contribute to poor biopsychosocial health. The conceptualization of OBS as trauma‐related stressors is aligned with decades of research on racism‐based stress theories that have conceptualized experiences of racism as traumatic stressors that lead to the development of PTSD (Carter, 2007; Williams et al., 2018). Among the extant literature, OBS have been found to serve as risk factors for PTSD symptomatology (Keating & Muller, 2020) and suicide behaviors (Green et al., 2022) for queer adolescents. However, much of the prior OBS research has primarily focused on one axis of oppression (e.g., heterosexism; Green et al., 2022; Keating & Muller, 2020), thus ignoring the unique experiences of intersectional OBS.

Queer BIPOC adolescents experience intersectional OBS that exist at the converging systems of heterosexism and racism, thus creating multidimensional stress experiences that may lead to PTSS and SITB. Intersectional OBS are associated with a host of negative mental health outcomes for queer BIPOC adolescents (Ching et al., 2024; Eisenberg et al., 2025; Mereish et al., 2022, 2023, 2025). Intersectional OBS faced by queer BIPOC communities manifest across multiple domains, including racism in lesbian, gay, bisexual, transgender, and queer (LGBTQ+) communities; cisheterosexism within one's own racial/ethnic community; and racism in dating and close relationships (Balsam et al., 2011). Cisheterosexism within one's own racial/ethnic community has been theorized as a particularly harmful intersectional OBS for queer BIPOC communities given that one's racial/ethnic community often serves as a major source of social support; therefore, identity invalidation from these networks can be particularly harmful.

Beyond identifying the risks of OBS, research must consider potential factors to aid in resilience and resistance against OBS and address the associations between OBS and poor mental health. Outness, defined as the extent to which an individual discloses their LGBTQ+ identity to others (Feinstein et al., 2022), is a multifaceted milestone that can function as a source of self‐affirmation and joy (Flynn et al., 2024; Hall et al., 2021). Simultaneously, outness can be a source of stress for queer adolescents in decision‐making related to coming out (Sullivan et al., 2024). Mixed research findings indicate that outness is associated with both positive and negative mental health outcomes. For example, Rentería et al. (2023) found that outness was associated with higher well‐being for gay and lesbian youth, yet it was associated with negative mental health outcomes for questioning youth. Other work demonstrated an association between higher degrees of outness and a higher likelihood of suicide attempts among LGBTQ+ youth (Feinstein et al., 2022). Given the importance of outness as a developmental milestone, it may serve as a moderator in the associations among intersectional OBS, PTSS, and SITB for queer BIPOC adolescents.

The present study aimed to examine the associations between intersectional OBS and PTSS, as well as the associations between intersectional OBS and SITB, in a sample of queer BIPOC adolescents, while exploring identity outness as a potential moderator in these associations. We hypothesized that intersectional OBS would be associated with higher PTSS and SITB (i.e., past‐year suicidal ideation, suicide planning, suicide attempts, and NSSI). Additionally, we hypothesized that identity outness would moderate these associations such that higher levels of identity outness would attenuate the associations between intersectional OBS and our outcomes (i.e., PTSS and SITB outcomes).

## METHOD

### Participants

Participants (*N* = 1,009) were 14–17 years of age (*M* = 15.97 years, *SD* = 0.93). A majority of participants identified as non‐White Hispanic/Latiné (36.7%), bisexual/pansexual/plurisexual (50.1%), cisgender girls (62.0%). A diverse range of racial/ethnic identities were reported: 21.5% identified as multiracial; 19.7% as Black or African American; 16.3% as Asian American or Pacific Islander; and 5.9% as Native American, American Indian, or Alaska Native. Detailed demographic information is presented in Supplementary Table S1.

### Procedure

The present study is a secondary data analysis using data derived from a parent study examining the effects of OBS on the well‐being of queer adolescents. The parent study featured a national sample (*N* = 2,558) of cisgender, queer adolescents who were recruited through social media outreach and respondent‐driven sampling methods between 2018 and 2019 (Schrager et al., 2022). Inclusion criteria were (a) aged 14–17 years, (b) identified as queer (i.e., lesbian, gay, bisexual, questioning, asexual, queer), (c) resided within the United States, and (d) identified as cisgender. Eligible participants were screened for potential fraudulent activity and identified as fraudulent based on poor data quality indicators (i.e., low survey duration time, failure to respond to attention checks, multiple survey attempts, duplicate internet protocol addresses and/or contact information, and a high decline‐to‐answer response rate). Responses from individuals identified as fraudulent were removed from the dataset. All study procedures were reviewed by the Social–Behavioral Institutional Review Board at the University of Southern California. Additional details regarding the study protocol have been previously described (Schrager et al., 2022). For the current study, data were analyzed for the subset of participants who identified as both queer and BIPOC (*N* = 1,009).

### Measures

#### Intersectional OBS

Intersectional OBS—specifically, cisheterosexism within one's racial/ethnic community—were measured using the three‐item Intersectionality subscale from the Sexual Minority Adolescent Stress Inventory (SMASI; Schrager et al., 2018). The SMASI asks participants to report on experiences of discrimination in their lifetime with “yes” or “no” response options. If a respondent endorses lifetime exposure to a stressor, they are asked to indicate (“yes” or “no”) whether they have experienced the stressor within the past 30 days. The SMASI Intersectionality subscale has demonstrated high reliability in previous research (e.g., Cronbach's α = .88; Schrager et al., 2024). Total lifetime intersectional OBS scores were summed and demonstrated acceptable reliability in the present sample, Cronbach's α = .70.

#### PTSS

PTSS were assessed using the abbreviated, six‐item PTSD Checklist (PCL‐6; Lang et al., 2012), which is derived from the longer PTSD Checklist–Civilian Version (Weathers et al., 1994). The PCL‐6 is based on the PTSD criteria outlined in the *Diagnostic and Statistical Manual of Mental Disorders* (4th ed.; *DSM‐IV*; American Psychiatric Association, 1994) and asks participants to report symptoms on a Likert scale ranging from 1 (*not at all*) to 5 (*extremely*). The PCL‐6 has demonstrated high reliability in previous samples (e.g., Cronbach's α = .78) and is a valid indicator for assessing PTSS. The scale was summed and demonstrated good reliability within our sample, Cronbach's α = .84. Given that we did not assess whether symptoms reported were related to a stressor that met *DSM‐IV* PTSD Criterion A, we characterized the responses as PTSS instead of PTSD symptoms.

#### SITB

Past‐year SITB and NSSI were assessed using questions from the national 2011 Youth Risk Behavior Survey (Centers for Disease Control and Prevention, 2011). Suicidal ideation (“During the past 12 months, did you ever seriously consider attempting suicide?”) and suicide planning (“During the past 12 months, did you make a plan about how you would attempt suicide?”) were assessed with one question each, with response options of “yes,” “no,” or “decline to answer.” Suicide attempts (“During the past 12 months, how many times did you actually attempt suicide?”) and NSSI (“During the past 12 months, how many times did you do something to purposely hurt yourself without wanting to die, such as cutting or burning yourself on purpose?”) were assessed with one question each with response options ranging from *0* to *6 or more times*. Given the nested nature of the suicide variables, participants were classified into four mutually exclusive groups based on their responses, consistent with prior research (Romanelli et al., 2022). The groups included: (a) no endorsement of suicidal ideation, plan, or attempts (*none* group); (b) the endorsement of suicidal ideation only (*ideation* group); (c) the endorsement of suicidal ideation and planning but no attempts (*ideation and plan* group); and (d) the endorsement of suicidal ideation, planning, and attempts (*all* group). NSSI was dichotomized into 0 (no endorsement) or 1 (one or more NSSI behaviors).

#### Identity outness

Identity outness was assessed using a scale developed by the parent study's researchers to measure disclosure of sexual identity (Schrager et al., 2022). Participants were asked, “Do the following people know that you are LGBTQ?,” with response options spanning family members, peers, educators, and other social agents. The scale was summed and demonstrated high reliability within our sample, Cronbach's α = .83.

#### Stress and sociodemographic variables

General stress was measured using the Perceived Stress Scale (PSS; Cohen et al., 1983), which asks participants to report on their past‐month experiences of stress. Responses were rated on a Likert scale ranging from 0 (*never*) to 4 (*very often*), with overall scale scores summed. Prior work has found the PSS to have high validity and reliability (Cronbach's αs > .70; Lee, 2012), which is consistent with the reliability within our sample, Cronbach's α = .80.

The question, “What is your race/ethnicity?” was used to assess participant racial/ethnic identity (i.e., Native American/American Indian/Alaska Native, Asian/Pacific Islander, Black or African American, White/Caucasian, Latino/Hispanic, multiracial, and race/ethnicity not listed here). Participants’ responses were recoded into six racial/ethnic categories, with participants identifying as White/Caucasian only excluded from the present analyses. Sexual identity, gender identity, age, socioeconomic status, and zip code were also assessed. Gender identity was assessed following the recommended two‐step approach of separately assessing sex assigned at birth and current gender identity (Bauer et al., 2017). Participants’ reported zip codes were dichotomized into “rural” or “urban” based on established rural–urban commuting area codes (Cromartie, 2019).

### Data analysis

All analyses were conducted using R (Version 4.4.2). Missing data within the entire sample at the measure level were minimal and ranged from 0.0% to 6.05%. For missing data within the intersectional OBS, identity outness, PCL‐6, and PSS scales, mean imputation was used to calculate the average of nonmissing response items for up to 30% of missing scale items, consistent with prior work conducted with queer BIPOC adolescents (Mereish et al., 2025). Our main hypotheses were tested using hierarchical linear, logistic, and multinomial regressions, featuring a noninteraction step and an interaction step for each outcome. In the first step, associations were examined among intersectional OBS, outness, and PTSS while accounting for perceived stress and demographic covariates. In the second step, identity outness was added as a moderator (i.e., the interaction between outness and intersectional OBS) to the linear regression model with PTSS as the outcome. Similarly, a multinomial logistic regression model with OBS and outness was conducted to assess the likelihood of being in one of the three suicide outcome groups (ideation only, ideation and plan, and all groups, with the none group serving as the reference). Finally, a hierarchical logistic regression model was conducted with OBS and identity outness as predictors for NSSI. Consistent with standard moderation analysis practices (Hayes, 2013), intersectional OBS and identity outness were mean‐centered to enhance interpretability and reduce multicollinearity between the variables. Due to the small sample sizes for each racial/ethnic group, there was insufficient statistical power to conduct subgroup analyses to examine differences in associations between intersectional OBS, outness, and PTSS and SITB for each race/ethnicity.

## RESULTS

Most participants reported intersectional OBS exposure in their lifetime (74.9%) and in the past 30 days (63.8%), as well as a high proportion of PTSS (69.8%). Nearly all participants (97.5%) reported disclosing their sexual identity to at least one person in their lives. For past‐year SITB, 42.4% of participants reported suicidal ideation, 27.4% reported suicide planning, and 16.1% made one or more suicide attempts. In recategorizing the suicide variables into distinctive groups, 52.9% of participants reported no suicidal ideation, planning, or attempts (none group); 11.4% had suicidal ideations but no further plan or attempts (ideation only group); 12.8% had suicidal ideations and created a plan but made no attempts (ideation and plan group); and 13.8% had suicidal ideations, created a plan, and attempted (all group). Finally, 38.0% of participants reported one or more experiences of NSSI.

As detailed in Table 1 (Step 1 of the model), linear regression analyses indicated that the overall main effects model was significant, *F*(15, 869) = 48.33, *p* < .001, *R* ^2^ = .45, and indicated that a greater number of lifetime exposures to intersectional OBS, β *=* .08, *p* = .002, and higher levels of outness, β = .08, *p* = .006, were significantly associated with greater past‐month PTSS after accounting for perceived stress and sociodemographic variables. Within the second step of the models (see Table 1, Step 2), analyses indicated that outness did not serve as a moderator of the association between intersectional OBS and PTSS symptomatology, β = .01, *p* = .621. Though the interaction model itself was significant, *F*(16, 868) = 45.29, *p* < .001, *R* ^2^ = .46, the incremental variance explained by the interaction model was minimal.

**TABLE 1:** Moderation analyses for posttraumatic stress symptoms (PTSS), suicide behaviors, and nonsuicidal self‐injury (NSSI) predicted by intersectional oppression‐based stress and outness.

	PTSS	Suicidal ideation only	Suicidal ideation and plan	Suicidal ideation, plan, and attempts	NSSI
Variable	β	a*OR*	a*OR*	a*OR*	a*OR*
Step 1 (noninteraction model)					
Oppression‐based stressors	.08^**^	1.13	1.39^**^	1.35^**^	1.22^*^
Outness	.08^**^	1.21	1.09	1.55^***^	1.21^*^
Perceived stress	.64^***^				
Step 2 (interaction model)					
Oppression‐based stressors	.08^**^	1.15	1.39^**^	1.33^*^	1.20^*^
Outness	.08^**^	1.20	1.08	1.55^***^	1.22^*^
Perceived stress	.64^***^				
Oppression‐Based Stressors x Outness	.01	0.86	1.10	1.04	1.14

*Note*: Steps 1 and 2 include the following covariates: racial/ethnic identity, sexual identity, gender identity, age, urbanicity, and free/reduced lunch status. Linear regression analyses were conducted with PTSS as the primary outcome. Multinomial regression analyses were conducted for the suicide outcome variables. None (no endorsements of suicidal ideation, planning, or attempts) served as the reference group for the multinomial suicide outcomes analyses. Logistic regression was conducted for the NSSI variable. PTSS = posttraumatic stress symptoms; NSSI = nonsuicidal self‐injury behaviors; a*OR* = adjusted odds ratio

* *p* < .05. **p < .01. ***p < .001.

Multinomial regression analyses were conducted to examine associations among lifetime intersectional OBS, outness, and suicide outcome groups. The main‐effects model (Table 1; Step 1) was significant χ^2^(45, *N* = 1,009) = 111.76, *p* < .001, Nagelkerke *R* ^2^ = .15, and revealed that a greater number of lifetime intersectional OBS were significantly associated with a higher likelihood of categorization in two suicide outcome groups: the ideation and plan group, adjusted odds ratio (a*OR*) *=* 1.39, 95% confidence interval (CI) [1.10, 1.75], and the all group, a*OR* = 1.35, 95% CI [1.09, 1.66]. Outness to a greater number of individuals was only associated with categorization in the all group, a*OR* = 1.55, 95% CI [1.22, 1.97]. Moderation analyses (Table 1; Step 2) indicated that the interaction model was nonsignificant in comparison to the main‐effects model, χ^2^(48, *N* = 1,009) = 2.89, *p* = .41, Nagelkerke *R* ^2^ = .15. Outness did not serve as a moderator in the associations between intersectional OBS and three suicide outcomes: ideation only: a*OR* = 0.86, 95% CI [0.68, 1.08]; ideation and plan: a*OR* = 1.10, 95% CI [0.87, 1.39]; and all, a*OR* = 1.04, 95% CI [0.82, 1.31].

The NSSI logistic regression main effects model was significant, χ^2^(15, *N* = 1,009) = 72.96, *p* < .001, Nagelkerke *R* ^2^ = .12, and demonstrated that higher levels of intersectional OBS, a*OR* = 1.22, 95% CI [1.05, 1.43], and identity outness, a*OR* = 1.21, 95% CI [1.02, 1.43], were associated with a higher likelihood of NSSI. Within the second step of the models (Table 1; Step 2), outness did not serve as a moderator, a*OR* = 1.14, 95% CI [0.97, 1.34], and the interaction model was not significant in comparison to the main‐effects model, χ^2^(16, *N* = 1,009) = 2.64, *p* = .10. Linear, multinomial, and logistic regression analyses were also assessed for past–30‐day experiences of intersectional OBS. The results showed the same patterns of significance for the associations among intersectional OBS, outness, PTSS, and SITB (see Supplementary Table S2).

## DISCUSSION

The current study examined the associations among intersectional OBS, PTSS, and SITB among queer BIPOC adolescents and tested the moderating role of identity outness on these associations. The findings from this study indicate that a greater number of intersectional OBS and a higher degree of identity outness were significantly associated with higher levels of PTSS. Furthermore, both greater exposure to intersectional OBS and a higher degree of identity outness were associated with a higher likelihood of endorsing suicidal ideation and engaging in suicide behaviors (i.e., endorsing suicidal ideation, suicide planning, and suicide attempts) and NSSI.

Our findings underscore that intersectional OBS function as significant stressors that may contribute to the development of trauma‐related symptoms among queer BIPOC adolescents. The associations observed among OBS, PTSS, and SITB are consistent with intersectional OBS and trauma‐informed frameworks positing that chronic exposure to oppression constitutes a form of complex stress that leads to PTSS (Carter, 2007; Mereish, 2024; Williams et al., 2018). These findings contribute to the growing body of literature documenting the adverse effects of OBS on the mental health of queer youth (Mereish et al., 2022; Roberts et al., 2012), highlighting intersectional OBS as a significant psychosocial stressor associated with PTSS and SITB among queer BIPOC adolescents.

Our findings for this specific sample of queer BIPOC adolescents indicate that outness itself can be a significant stressor that is associated with PTSS and high levels of suicide behaviors and NSSI. This supports prior research indicating that navigating identity disclosure can be a developmentally stressful process (Sullivan et al., 2024) that is associated with self‐harm (Feinstein et al., 2022). However, it is important to note that outness also fosters positive and joyful developmental outcomes, such as increased affirming social support, validation, and a stronger sense of self (Flynn et al., 2024), thus highlighting strength, resilience, and resistance in queer youth (Kosciw et al., 2015). Therefore, these findings support prior research that theorizes outness as a multifaceted cognitive–affective–behavioral construct (Brennan et al., 2021), and our measure may not have fully captured this complexity. Future research should utilize more comprehensive assessments of outness that consider context, internal processes, and environmental safety. Though outness demonstrated its own significant associations with mental health outcomes in this study, it did not buffer or amplify the negative associations between intersectional OBS and PTSS or SITB within our sample. This finding indicates that the benefits or risks associated with outness might be operating somewhat independently of the associations of OBS within this sample.

Although our findings highlight nuanced associations between intersectional OBS and outness and mental health outcomes among queer BIPOC adolescents, there are some important limitations. A primary study limitation was the focus on cisgender adolescents, which limits capturing the nuanced OBS experiences of transgender and gender‐diverse adolescents. Additionally, participants in our sample lived in predominantly urban locations, which may limit the generalizability of the findings to rural queer adolescents. Lower participation from rural adolescents may be due to factors such as increased stigma or fewer LGBTQ+ community resources. Future studies should specifically engage rural queer BIPOC adolescents to examine how geographic contexts may shape exposure to OBS and related mental health outcomes. Although our intersectional OBS measure highlighted the nuances of experiencing cisheterosexism from one's own racial/ethnic community, the measure unfortunately does not capture other highly correlated experiences of OBS, including cisheterosexism and racism from outside one's racial/ethnic community. Furthermore, the SMASI's format for assessing exposure does not differentiate between one‐time and chronic stress exposures, potentially grouping adolescents who experienced OBS once with those who experienced it frequently, therefore affecting the noted associations.

Our findings suggest that intersectional OBS serve as prominent stressors linked significantly to PTSS and SITB among queer BIPOC adolescents. Given that identity outness did not attenuate the associations among OBS, PTSS, and SITB, further longitudinal research is needed to identify the unique role of outness among queer BIPOC adolescents. Finally, future research must prioritize the nuanced experiences of queer BIPOC adolescents to inform culturally affirming and trauma‐informed interventions and policies that directly address systemic oppressions as a source of harm, thus promoting affirming and protective environments for this multiply oppressed population (Mereish, 2024).

## AUTHOR NOTE

This work was supported by the National Institute on Minority Health and Health Disparities (R01MD012252).

The content is solely the responsibility of the authors and does not necessarily represent the official views of the National Institutes of Health. We have no conflicts of interest to disclose.

We are extremely grateful for the life, legacy, and contributions of Dr. Jeremy Goldbach, who sadly passed away on June 7, 2025. Dr. Goldbach's impact on research supporting the health and well‐being of queer and transgender adolescents, as well as his impact on his colleagues, mentees, friends, family, and community, cannot be understated. This manuscript is in his honor.

## OPEN PRACTICES STATEMENT

The studies reported in this article (parent and secondary data analysis) were not formally preregistered. Neither the data nor the materials have been made available on a permanent third‐party archive. The data that support the findings of this study are available from the corresponding author upon reasonable request at emereish@umd.edu.

## AUTHOR CONTRIBUTIONS

**Tara R. Sullivan**: conceptualization, investigation, writing—original draft, writing—review and editing, formal analysis, visualization. **Marianna Amato**: writing—original draft, writing—review and editing. **Jeremy T. Goldbach**: conceptualization, funding acquisition, writing—review and editing, project administration, data curation, supervision, methodology, investigation. **Ethan H. Mereish**: conceptualization, investigation, writing—original draft, writing—review and editing, supervision.

## Supporting information



SUPPORTING INFORMATION

## References

[jts70069-bib-0001] American Psychiatric Association . (1994). Diagnostic and statistical manual of mental disorders (4th ed.). Author.

[jts70069-bib-0002] Bauer, G. R. , Braimoh, J. , Scheim, A. I. , & Dharma, C. (2017). Transgender‐inclusive measures of sex/gender for population surveys: Mixed‐methods evaluation and recommendations. PLoS One, 12(5), Article e0178043. 10.1371/journal.pone.0178043 28542498 PMC5444783

[jts70069-bib-0003] Balsam, K. F. , Molina, Y. , Beadnell, B. , Simoni, J. , & Walters, K. (2011). Measuring multiple minority stress: The LGBT People of Color Microaggressions Scale. Cultural Diversity & Ethnic Minority Psychology, 17(2), 163–174. 10.1037/a0023244 21604840 PMC4059824

[jts70069-bib-0004] Bostwick, W. B. , Meyer, I. , Aranda, F. , Russell, S. , Hughes, T. , Birkett, M. , & Mustanski, B. (2014). Mental health and suicidality among racially/ethnically diverse sexual minority youths. American Journal of Public Health, 104(6), 1129–1136. 10.2105/AJPH.2013.301749 24825217 PMC4062032

[jts70069-bib-0005] Brennan, J. M. , Dunham, K. J. , Bowlen, M. , Davis, K. , Ji, G. , & Cochran, B. N. (2021). Inconcealable: A cognitive–behavioral model of concealment of gender and sexual identity and associations with physical and mental health. Psychology of Sexual Orientation and Gender Diversity, 8(1), 80–93. 10.1037/sgd0000424

[jts70069-bib-0006] Carter, R. T. (2007). Racism and psychological and emotional injury: Recognizing and assessing race‐based traumatic stress. The Counseling Psychologist, 35(1), 1–93. 10.1177/0011000006292033

[jts70069-bib-0007] Centers for Disease Control and Prevention . (2011). Youth Risk Behavior Survey . https://www.cdc.gov/yrbs/data/national‐yrbs‐datasets‐documentation.html

[jts70069-bib-0008] Ching, T. H. W. , Finkelstein‐Fox, L. , Lee, S. Y. , & Watson, R. J. (2024). Effects of sexual and gender minority stress on depressive symptoms among adolescents of color in the United States. Cultural Diversity & Ethnic Minority Psychology, 30(2), 309–318. 10.1037/cdp0000562 36048116 PMC9975117

[jts70069-bib-0009] Cohen, S. , Kamarck, T. , & Mermelstein, R. (1983). A global measure of perceived stress. Journal of Health and Social Behavior, 24(4), 385–396. 10.2307/2136404 6668417

[jts70069-bib-0010] Crenshaw, K. (1989). Demarginalizing the intersection of race and sex: A Black feminist critique of antidiscrimination doctrine, feminist theory, and antiracist politics. University of Chicago Legal Forum, 1989, *1*, Article 8. http://chicagounbound.uchicago.edu/uclf/vol1989/iss1/8

[jts70069-bib-0011] Cromartie, J. (2019). Rural–urban commuting area codes . Economic Research Service. https://www.ers.usda.gov/data‐products/rural‐urban‐commuting‐area‐codes

[jts70069-bib-0012] Eisenberg, M. E. , Watson, R. J. , Lawrence, S. E. , Simon, K. , Brightly‐Brown, S. , Mereish, E. H. , Wright, M. N. , & Rider, G. N. (2025). Intersectional microaggressions among LGBTQ+ youth of color: How are they associated with well‐being? Annals of LGBTQ Public and Population Health, 6(4), 392–407. 10.1891/lgbtq-2024-0020

[jts70069-bib-0013] Ellis, É. M. , & Tate, A. (2022). Is trauma exposure more harmful for sexual minority youth? Differences in trauma‐suicide associations in a nationally representative sample of U.S. youth and implications for suicide prevention. Journal of Child & Adolescent Trauma, 16(2), 173–182. 10.1007/s40653-022-00475-0 37234833 PMC10205931

[jts70069-bib-0014] Feinstein, B. A. , Mereish, E. H. , Mamey, M. R. , Chang, C. J. , & Goldbach, J. T. (2022). Age differences in the associations between outness and suicidality among LGBTQ+ youth. Archives of Suicide Research, 27(2), 734–748. 10.1080/13811118.2022.2066493 35506502 PMC9719400

[jts70069-bib-0015] Flynn, S. S. , Touhey, S. , Sullivan, T. R. , & Mereish, E. H. (2024). Queer and transgender joy: A daily diary qualitative study of positive identity factors among sexual and gender minority adolescents. *Psychology of Sexual Orientation and Gender Diversity*. Advance online publication. 10.1037/sgd0000733 PMC12377554 40881959

[jts70069-bib-0016] Green, A. E. , Price, M. N. , & Dorison, S. H. (2022). Cumulative minority stress and suicide risk among LGBTQ youth. American Journal of Community Psychology, 69(1–2), 157–168. 10.1002/ajcp.12553 34534356

[jts70069-bib-0017] Hall, W. J. , Dawes, H. C. , & Plocek, N. (2021). Sexual orientation identity development milestones among lesbian, gay, bisexual, and queer people: A systematic review and meta‐analysis. Frontiers in Psychology, 12, Article 753954. 10.3389/fpsyg.2021.753954 34777153 PMC8581765

[jts70069-bib-0018] Hayes, A. F. (2013). Introduction to mediation, moderation, and conditional process analysis: A regression‐based approach (3rd ed.). The Guilford Press.

[jts70069-bib-0019] Keating, L. , & Muller, R. T. (2020). LGBTQ+ based discrimination is associated with PTSD symptoms, dissociation, emotion dysregulation, and attachment insecurity among LGBTQ+ adults who have experienced trauma. Journal of Trauma & Dissociation, 21(1), 124–141. 10.1080/15299732.2019.1675222 31581904

[jts70069-bib-0020] Kosciw, J. G. , Palmer, N. A. , & Kull, R. M. (2015). Reflecting resiliency: Openness about sexual orientation and/or gender identity and its relationship to well‐being and educational outcomes for LGBT students. American Journal of Community Psychology, 55(1–2), 167–178. 10.1007/s10464-014-9642-6 24691967

[jts70069-bib-0021] Lang, A. J. , Wilkins, K. , Roy‐Byrne, P. P. , Golinelli, D. , Chavira, D. , Sherbourne, C. , Rose, R. D. , Bystritsky, A. , Sullivan, G. , Craske, M. G. , & Stein, M. B. (2012). Abbreviated PTSD Checklist (PCL) as a guide to clinical response. General Hospital Psychiatry, 34(4), 332–338. 10.1016/j.genhosppsych.2012.02.003 22460001 PMC3383936

[jts70069-bib-0022] Lee, E.‐H. (2012). Review of the psychometric evidence of the Perceived Stress Scale. Asian Nursing Research, 6(4), 121–127. 10.1016/j.anr.2012.08.004 25031113

[jts70069-bib-0023] Mereish, E. H. (2024). Oppression‐based stress and alcohol inequities among sexual and gender minority people: An intersectional multilevel framework. Alcohol Research: Current Reviews, 44(1), Article 05. 10.35946/arcr.v44.1.05 39246430 PMC11379061

[jts70069-bib-0024] Mereish, E. H. , Abramson, J. R. , Lee, H. , & Watson, R. J. (2025). Intersectional oppression‐based stress, drinking to cope motives, and alcohol use and hazardous drinking among sexual and gender minority adolescents who are Black, Indigenous, and people of color. LGBT Health, 12(2), 125–133. 10.1089/lgbt.2024.0023 38973422 PMC12021789

[jts70069-bib-0025] Mereish, E. H. , Fish, J. N. , & Watson, R. J. (2023). Intersectional minority stress and alcohol, tobacco, and cannabis use among sexual and gender minority adolescents of color: Moderating role of family support. LGBT Health, 10(1), 18–25. 10.1089/lgbt.2021.0430 35914084 PMC10024056

[jts70069-bib-0026] Mereish, E. H. , Parra, L. A. , Watson, R. J. , & Fish, J. N. (2022). Subtle and intersectional minority stress and depressive symptoms among sexual and gender minority adolescents of color: Mediating role of self‐esteem and sense of mastery. Prevention Science, 23(1), 142–153. 10.1007/s11121-021-01294-9 34482516 PMC9802675

[jts70069-bib-0027] Rentería, R. , Feinstein, B. A. , Dyar, C. , & Watson, R. J. (2023). Does outness function the same for all sexual minority youth? Testing its associations with different aspects of well‐being in a sample of youth with diverse sexual identities. Psychology of Sexual Orientation and Gender Diversity, 10(3), 490–497. 10.1037/sgd0000547 37873023 PMC10593417

[jts70069-bib-0028] Roberts, A. L. , Rosario, M. , Corliss, H. L. , Koenen, K. C. , & Austin, S. B. (2012). Elevated risk of posttraumatic stress in sexual minority youths: Mediation by childhood abuse and gender nonconformity. American Journal of Public Health, 102(8), 1587–1593. 10.2105/AJPH.2011.300530 22698034 PMC3395766

[jts70069-bib-0029] Rogers, M. L. , & Taliaferro, L. A. (2020). Self‐injurious thoughts and behaviors among sexual and gender minority youth: A systematic review of recent research. Current Sexual Health Reports, 12(4), 335–350. 10.1007/s11930-020-00295-z

[jts70069-bib-0030] Romanelli, M. , Sheftall, A. H. , Irsheid, S. B. , Lindsey, M. A. , & Grogan, T. M. (2022). Factors associated with distinct patterns of suicidal thoughts, suicide plans, and suicide attempts among U.S. adolescents. Prevention Science, 23(1), 73–84. 10.1007/s11121-021-01295-8 34482517 PMC8792183

[jts70069-bib-0031] Schrager, S. M. , Goldbach, J. T. , & Mamey, M. R. (2018). Development of the Sexual Minority Adolescent Stress Inventory. Frontiers in Psychology, 9, Article 319. 10.3389/fpsyg.2018.00319 29599737 PMC5862853

[jts70069-bib-0032] Schrager, S. M. , Mamey, M. R. , Rhoades, H. , & Goldbach, J. T. (2022). Adolescent stress experiences over time study (ASETS) protocol: Design and methods of a prospective longitudinal study of sexual minority adolescents in the USA. BMJ Open, 12(3), Article e054792. 10.1136/bmjopen-2021-054792 PMC8915334 35264352

[jts70069-bib-0033] Schrager, S. M. , Mamey, M. R. , Rusow, J. , & Goldbach, J. T. (2024). Development and preliminary validation of the Sexual Minority Adolescent Stress Inventory–Short Form. *Psychology of Sexual Orientation and Gender Diversity*. Advance online publication. 10.1037/sgd0000706 PMC12373156 40857465

[jts70069-bib-0034] Sullivan, T. R. , Flynn, S. S. , Touhey, S. , & Mereish, E. H. (2024). Through their eyes: A qualitative, daily diary exploration of oppression‐based stress experiences among sexual and gender minority adolescents. *Psychology of Sexual Orientation and Gender Diversity*. Advance online publication. 10.1037/sgd0000779 PMC12499921 41059482

[jts70069-bib-0035] Weathers, F. , Litz, B. , Huska, J. , & Keane, T. (1994). PTSD Checklist for DSM‐IV–Civilian Version (PCL‐C) . National Center for PTSD, Behavioral Science Division. https://www.ptsd.va.gov/professional/assessment/documents/APCLC.pdf

[jts70069-bib-0036] Williams, M. T. , Metzger, I. W. , Leins, C. , & DeLapp, C. (2018). Assessing racial trauma within a *DSM–5* framework: The UConn Racial/Ethnic Stress & Trauma Survey. Practice Innovations, 3(4), 242–260. 10.1037/pri0000076

