# Supplementary material for: Oppression‐based stressors, posttraumatic stress symptoms, and self‐injurious thoughts and behaviors among a national sample of queer adolescents of color
Source: J Trauma Stress. 2026 Apr 22;39(4):655–62. doi: 10.1002/jts.70069 (PMC13436484; doi:10.1002/jts.70069)
Supplement: Supplementary file 1 [file JTS-39-655-s001.docx]

*Supplementary Table S1. Participant Characteristics by Race/Ethnicity*

|  | **Total** | **Asian** | **Black/** | **Multiracial** | **Native** | **Non-White** |
| --- | --- | --- | --- | --- | --- | --- |
|  | **Sample** | **American** | **African** | **(*n* = 217)** | **American/American** | **Latiné** |
|  | **(*N* = 1,009)** | **or Pacific** | **American** |  | **Indian/Alaska** | **(*n* = 370)** |
|  |  | **Islander** | **(*n* = 199)** |  | **Native** |  |
|  |  | **(*n* = 164)** |  |  | **(*n* = 59)** |  |
| **Age (Years)** |  |  |  |  |  |  |
| **Mean (SD)** | 15.97 | 16.02 | 16.04 | 15.89 | 15.92 | 15.98 |
|  | (0.93) | (0.86) | (0.91) | (0.96) | (0.95) | (0.96) |
|  | ***N* (%)** | ***N* (%)** | ***N* (%)** | ***N* (%)** | ***N* (%)** | ***N* (%)** |
| **Sexual Identity** |  |  |  |  |  |  |
| **Lesbian** | 180 | 26 | 40 | 37 | 11 | 66 |
|  | (17.84%) | (15.85%) | (20.10%) | (17.05%) | (18.64%) | (17.84%) |
| **Gay** | 262 | 43 | 39 | 51 | 14 | 115 |
|  | (25.97%) | (26.22%) | (19.60%) | (23.50%) | (23.73%) | (31.08%) |
| **Bisexual/Pansexual** | 475 | 79 | 99 | 104 | 29 | 164 |
|  | (47.08%) | (48.17%) | (49.75%) | (47.93%) | (49.15%) | (44.32%) |
| **Queer** | 30 | 6 | 5 | 9 | 1 | 9 |
|  | (2.97%) | (3.66%) | (2.51%) | (4.15%) | (1.69%) | (2.43%) |
| **Another Sexual** | 62 | 10 | 16 | 16 | 4 | 16 |
| **Identity** | (6.14%) | (6.10%) | (8.04%) | (7.37%) | (6.78%) | (4.32%) |
| **Gender Identity** |  |  |  |  |  |  |
| **Cisgender girl** | 626 | 99 | 136 | 142 | 39 | 210 |
|  | (62.04%) | (60.37%) | (68.34%) | (65.44%) | (66.10%) | (56.76%) |
| **Cisgender boy** | 383 | 65 | 63 | 75 | 20 | 160 |
|  | (37.96%) | (39.63%) | (31.66%) | (34.56%) | (33.90%) | (43.24%) |
| **Sex Assigned at Birth** |  |  |  |  |  |  |
| **Female** | 626 | 99 | 136 | 142 | 39 | 210 |
|  | (62.04%) | (60.36%) | (68.34%) | (65.44%) | (66.10%) | (56.76%) |
| **Male** | 383 | 65 | 63 | 75 | 20 | 160 |
|  | (37.96%) | (39.63%) | (31.66%) | (34.56%) | (33.90%) | (43.24%) |
| **Free/Reduced Lunch** |  |  |  |  |  |  |
| **Receives** | 529 | 47 | 128 | 82 | 34 | 238 |
| **Free/Reduced**  **Lunch** | (52.43%) | (28.66%) | (64.32%) | (37.79%) | (57.63%) | (64.32%) |
| **Does not receive** | 356 | 104 | 50 | 102 | 18 | 82 |
| **Free/Reduced** | (35.28%) | (63.41%) | (25.13%) | (47.00%) | (30.51%) | (22.16%) |
| **Lunch**  **Urbanicity** |  |  |  |  |  |  |
| **Rural** | 109 | 9 | 16 | 28 | 20 | 36 |
|  | (10.80%) | (5.49%) | (8.04%) | (12.90%) | (33.90%) | (9.73%) |
| **Urban** | 900 | 155 | 183 | 189 | 39 | 334 |
|  | (89.20%) | (94.51%) | (91.96%) | (87.10%) | (66.10%) | (90.27%) |
| **Oppression-Based Stress (OBS)** |  |  |  |  |  |  |
| **Lifetime OBS** | 756 | 143 | 152 | 148 | 39 | 274 |
|  | (74.93%) | (87.20%) | (76.38%) | (68.20%) | (66.10%) | (74.05%) |
| **No lifetime OBS** | 253 | 21 | 47 | 69 | 20 | 96 |
|  | (25.07%) | (12.80%) | (23.62%) | (31.80%) | (33.90%) | (25.95%) |
| **Past 30-day OBS** | 644 | 125 | 138 | 121 | 34 | 226 |
|  | (63.83%) | (76.22%) | (69.35%) | (55.76%) | (57.63%) | (61.08%) |
| **No 30-day OBS** | 365 | 39 | 61 | 96 | 25 | 144 |
|  | (36.17%) | (23.78%) | (30.65%) | (44.24%) | (42.38%) | (38.92%) |
| **Outness** |  |  |  |  |  |  |
| **Has disclosed to at** | 985 | 157 | 191 | 214 | 57 | 365 |
| **least one person** | (97.62%) | (95.73%) | (95.98%) | (98.62%) | (96.61%) | (98.65%) |
| **No prior disclosures** | 25 | 7 | 2 | 3 | 2 | 5 |
|  | (2.48%) | (4.27%) | (1.01%) | (1.38%) | (3.39%) | (1.35%) |
| **PTSS Symptoms** |  |  |  |  |  |  |
| **High PTSS** | 704  (69.77%) | 108 (65.85%) | 142  (71.36%) | 152  (70.05%) | 45  (76.27%) | 257  (69.46%) |
| **Low PTSS** | 305  (30.23%) | 56  (34.14%) | 57  (28.64%) | 65  (29.95%) | 14  (23.73%) | 113  (30.54%) |
|  |  |  |  |  |  |  |
| **Past-year Suicide Ideation** |  |  |  |  |  |  |
| **Yes** | 428  (42.42%) | 51  (31.10%) | 92  (46.23%) | 103  (47.47%) | 26  (44.07%) | 156  (42.16%) |
|  |  |  |  |  |  |  |
| **No** | 534  (52.92%) | 103  (62.80%) | 100  (50.25%) | 103  (47.47%) | 29  (49.15%) | 199  (53.78%) |
|  |  |  |  |  |  |  |
| **Past-year Suicide**  **Plan** |  |  |  |  |  |  |
| **Yes** | 276  (27.35%) | 32  (19.51%) | 60  (30.15%) | 71  (32.72%) | 15  (25.42%) | 98  (26.49%) |
| **No** | 674  (66.80%) | 122  (74.39%) | 129  (64.82%) | 131  (60.37%) | 39  (66.10%) | 253  (68.38%) |
|  |  |  |  |  |  |  |
| **Past-year Suicide Attempt** |  |  |  |  |  |  |
| **Yes** | 162  (16.06%) | 10  (6.10%) | 38  (19.10%) | 36  (16.59%) | 13  (22.03%) | 65  (17.57%) |
| **No** | 786 (77.90%) | 143  (87.20%) | 150  (75.38%) | 168  (77.42%) | 42  (71.19%) | 283  (76.49%) |
| **Past-year NSSI** |  |  |  |  |  |  |
| **Yes** | 383  (37.96%) | 46  (28.05%) | 68  (34.17%) | 101  (46.54%) | 25  (42.37%) | 143  (38.65%) |
| **No** | 571  (56.59%) | 110 (67.07%) | 120  (60.30%) | 101  (46.54%) | 31  (52.54%) | 209  (56.49%) |
|  |  |  |  |  |  |  |
| **No Suicide Ideation or Behaviors (None Group)** | 534 (52.92%) | 103 (62.80%) | 100  (50.25%) | 103  (47.47%) | 29  (49.15%) | 199  (53.78%) |
|  |  |  |  |  |  |  |
| **Suicide Ideation (Ideation Only Group)** | 115 (11.40%) | 18  (10.98%) | 21  (10.55%) | 25  (11.52%) | 8  (13.56%) | 43  (11.62%) |
|  |  |  |  |  |  |  |
| **Suicide Ideation and Suicide Plan (Ideation and Plan Group)** | 129 (12.78%) | 22  (13.41%) | 27  (13.57%) | 37  (17.05%) | 4  (6.78%) | 39  (10.54%) |
|  |  |  |  |  |  |  |
| **Suicide Ideation, Plan, and Attempts (All Group)** | 139 (13.78%) | 9  (5.49%) | 31  (15.58%) | 32  (14.75%) | 11  (18.64%) | 56  (15.14%) |
|  |  |  |  |  |  |  |
| *Note.* SD = standard deviation, PTSS = posttraumatic stress symptoms, NSSI = non-suicidal self-injury. High PTSS is indicated with a score of 14 or higher on the PCL-C. | | | | | |  |
|  |  |  |  |  |  |  |
|  |  |  |  |  |  |  |

| *Supplementary Table S2. Moderation Analyses for Posttraumatic Stress Symptoms (PTSS), Suicide Behaviors, and Non-Suicidal Self-Injury (NSSI) Predicted by Past 30-Day Intersectional Oppression-Based Stress and Outness* | | | | | |
| --- | --- | --- | --- | --- | --- |
| *Variable* | *PTSS* | *Suicide Ideation Only* | *Suicide Ideation and Plan* | *Suicide Ideation, Plan, and Attempts* | *NSSI* |
| Step 1 (Non-Interaction Model) | *β* | *AOR* | *AOR* | *AOR* | *AOR* |
| Model Significance | *F(*15, 869) = 48.59  ******* | χ^2^(45, *N* = 1,009) = 115.20  ******* | | | χ^2^(15, *N* = 1,009) = 74.58  ******* |
| *R*^2^ and Nagelkerke *R*^2^ | .46 | .15 | | | .14 |
|  |  |  |  |  |  |
| Oppression-Based Stress | **.09**  ******* | 1.17 | **1.34**  * | **1.47**  ******* | **1.26**  ****** |
| Outness | **.08**  ****** | 1.20 | 1.05 | **1.57**  ******* | **1.24**  ***** |
| Perceived Stress | **.63**  ******* | **—** | **—** | **—** | **—** |
| Step 2 (Interaction Model) | *β* | *AOR* | *AOR* | *AOR* | *AOR* |
| Model Significance | *F(*16, 868) = 46.01  *** | χ^2^(48, *N* = 1,009) = 119.26  ******* | | | χ^2^(16, *N* = 1,009) = 80.89  ******* |
| *R*^2^ and Nagelkerke *R*^2^ | .46 | .15 | | | .15 |
| Model Comparison | χ^2^(18, *N* = 1,009) = 4.48  ***** | χ^2^(48, *N* = 1,009) = 4.06 | | | χ^2^(16, *N* = 1,009) = 5.65  ***** |
| Oppression-Based Stress | **.09**  ****** | 1.19 | **1.33**  ****** | **1.41**  ****** | **1.22**  ***** |
| Outness | **.08**  ****** | 1.20 | 1.04 | **1.56**  ******* | **1.24**  ****** |
| Perceived Stress | **.63**  ******* | **—** | **—** | **—** | **—** |
| Oppression-Based Stress ⋅ Outness | **.06**  ***** | 0.90 | 1.17 | 1.13 | **1.21**  ***** |
| *Note*. AOR = adjusted odds ratio. Steps 1 and 2 include the following covariates: racial/ethnic identity, sexual identity, gender identity, age, urbanicity, and free/reduced lunch status. None (no endorsements of suicide ideation, planning, or attempts) served as the reference group for the multinomial suicide outcomes analyses.  * *p* < 0.05 ; ** *p* < 0.01 ; *** *p* < 0.001 | | | | | |
